# Comparison of the In Vitro and Ex Vivo Permeation of Existing Topical Formulations Used in the Treatment of Facial Angiofibroma and Characterization of the Variations Observed

**DOI:** 10.3390/pharmaceutics12111060

**Published:** 2020-11-07

**Authors:** Guillaume Le Guyader, Bernard Do, Victoire Vieillard, Karine Andrieux, Muriel Paul

**Affiliations:** 1 Department of Pharmacy, AP-HP, Hôpital Henri Mondor, F-94010 Créteil, France; bernard.do@aphp.fr (B.D.); victoire.vieillard@aphp.fr (V.V.); muriel.paul@aphp.fr (M.P.); 2 Department of Pharmacy, CHI Creteil, F-94010 Créteil, France; 3 Department of Pharmacy, Université Paris-Saclay, Matériaux et Santé, 92296 Châtenay-Malabry, France; 4 Department of Pharmacy, Université de Paris, CNRS, INSERM, UTCBS, F-75006 Paris, France; karine.andrieux@parisdescartes.fr; 5 Department of Pharmacy, EpidermE, Université Paris Est Créteil, F-94010 Créteil, France

**Keywords:** semi-solid topical formulations, topical rapamycin, formulation parameters, permeation, Franz diffusion cells, Strat-M, in vitro permeation test, human skin, angiofibromas, tuberous sclerosis complex

## Abstract

Rapamycin has been used topically to treat facial angiofibromas associated with tuberous sclerosis for more than a decade. In the absence of a commercial form, a large number of formulations have been clinically tested. However, given the great heterogeneity of these studies, particularly with regard to the response criteria, it was difficult to know the impact and thus to compare the relevance of the formulations used. The objective of this work was therefore to evaluate the link between the diffusion of rapamycin and the physico-chemical characteristics of these different formulations on Strat-M^®^ membranes as well as on human skin using Franz cells. Our results underline the importance of the type of vehicle used (hydrogel > cream > lipophilic ointment), the soluble state of rapamycin and its concentration close to saturation to ensure maximum thermodynamic activity. Thus, this is the first time that a comparative study of the different rapamycin formulations identified in the literature for the management of facial angiofibromas has been carried out using a pharmaceutical and biopharmaceutical approach. It highlights the important parameters to be considered in the development and optimization of topical rapamycin formulations with regard to cutaneous absorption for clinical efficacy.

## 1. Introduction

Tuberous sclerosis complex (TSC) is an autosomal dominant disease caused by the constitutive activation of the mammalian target of rapamycin. TSC is characterized by excessive cellular growth and proliferation, causing multiple hamartomas that can affect different organs. Facial angiofibromas are the most emblematic skin manifestations of this pathology and occur in 80% of patients with TSC [1,2]. Angiofibromas often appear in early childhood and, although they are usually asymptomatic, they can be very disfiguring, affecting the patient both physically and psychologically with a major impact on Quality of Life [3]. Since 2010, randomized controlled trial or isolated case reports have been published and have shown the efficacy of topical rapamycin for angiofibromas linked to TSC particularly in children (70.9% of cases with a median age of 14.5 years), with generally mild or moderate local side effects reported [3,4,5,6,7]. Recently a systematic study with meta-analyses supported that topical rapamycin is effective in the management of angiofibromas linked to TSC [7]. However, as mentioned by the author, the results of the clinical studies cannot be pooled for a meta-analysis because of their heterogeneity, particularly with regard to the evaluation of the efficacy criterion. Indeed, many authors use a subjective criterion while few studies use an objective score, such as the Facial Angiofibroma Severity Index. Similarly, there are differences in the evolution of skin lesions (fibrotic or non-fibrotic tumors) associated with the age of the patient, suggesting better efficacy in younger patients [8].

Finally, to achieve rapamycin transdermal permeation and given the absence of a commercial topical form, due to the lack of demand, there is great variability in the type of formulations offered (solution, ointment, cream or gel), in the concentrations tested (0.003 to 1%), in the doses prescribed (once or twice a day or intermittently) and in the duration of treatment. It should also be noted that commercial oral liquid forms based on rapamycin were even used in the early stages for lack of other alternatives to meet a targeted and particularly critical need for the patient. It is therefore difficult, under these conditions, to compare these studies and deduce the most effective formulations in terms of diffusion. Indeed, most of the published studies focus on the observed pharmacological effect, but the penetration of the drug into the dermis is one of the main factors contributing to efficacy.

Percutaneous absorption of the active ingredient involves several complex steps including: (i) dissolution and release of the drug from its vehicle/formulation, (ii) drug partitioning and diffusion into the *stratum corneum*, mainly through intercellular pathways, and (iii) penetration into the deeper viable layers of the skin [9]. The diffusion of the active ingredient through the skin thus depends on the physiopathological characteristics of the skin, on the excipient formulation but also on the physico-chemical characteristics of the active ingredient [10]. In 1997, Lipinski conducted a retrospective study on 2245 drugs in order to identify the physico-chemical properties that predict the diffusion capacity of the active ingredient through biological membranes [11]. Four physical chemical characteristics must be taken into account, namely the drug molecular weight (M_w_) which determines the diffusion coefficient (D), the octanol-water partition coefficient that represents the *stratum corneum* partition coefficient (P), the number of hydrogen-bond donors (NHD) and of acceptors (NHA) that control the interactions with the surface of corneocytes [12,13]. Recently, Choy and Prausnitz concluded that low absorption or permeation through the skin is expected when M_w_ > 335 daltons, LogP > 5, NHD > 2 and NHA > 5 [13]. As a result, with a M_w_ of 914 daltons, a predictive LogP of 4.3 and a NHD and NHA of 3 and 13, respectively, for rapamycin, low permeation through the upper layers of the epidermis to the deep dermal layer would be significantly limited. Moreover, its low solubility in water (2.6 µg mL^−1^) as well as its ionized form (pKa 9.96) at skin pH (≈4.8) are important parameters that limit the passive diffusion of the molecule. The dosage form formulation and the excipients composition are therefore critical insofar as they could influence the diffusion and thus the efficacy of the active ingredient.

On the whole, it is commonly accepted that the physical state of the drug molecule (solubilized or dispersed), the type of vehicle (hydrophilic or lipophilic) as well as the concentration of active ingredient in the formulation are critical factors that influence percutaneous flow [14].

The objective of this work is therefore to characterize these different parameters in the existing formulations in order to compare and understand their respective behaviors in terms of rapamycin topical penetration performance. Thus, this comparative study was performed against two systems with different levels of complexity. Initially, in vitro tests on Strat-M^®^ synthetic membrane were carried out in order to assess the impact of the semi-solid base nature on the one hand and the solubilized or non-solubilized state of rapamycin on the other. For this purpose, the concentration of active ingredient was set at 0.1%, corresponding to the median value used in the various reports [7]. Then, the importance of the concentration of rapamycin was studied through Strat-M^®^ membranes, for concentrations ranging from 0.05 to 2% on the most suitable formulations. Finally, ex vivo tests on human skin with fixed concentration of rapamycin (0.1%), were carried out in order to mimic as much as possible the conditions of application in humans, to evaluate the distribution of rapamycin in each skin compartment (epidermis and dermis) and to compare the results obtained with synthetic membranes.

## 2. Materials and Methods

### 2.1. Rapamycin Formulations Tested

The five main formulations evaluated in literature were prepared as follows:

- Formulations 1 (F1) corresponding to rapamycin lipophilic ointment. F1 was prepared from rapamycin active pharmaceutical ingredient (API) (Inresa) dispersed in petroleum (Vaseline officinale^®^, Cooper) [4,5,15,16,17,18,19,20,21,22,23,24,25,26,27].
- Formulations 2 (F2) and 3 (F3) corresponding to rapamycin lipophilic/hydrophilic creams. F2 was prepared using rapamycin API dispersed in Dexeryl^®^ cream (F2) (Pierre Fabre) [8,28,29]. Dexeryl^®^ is composed mainly of water, glycerin (15%, *w*/*w*) and 10% of lipophilic phase (8% petroleum jelly and 2% paraffin oil, *w*/*w*). For F3, API was previously solubilized in 5% (*w*/*w*) of Transcutol^®^ P (Diethylene glycol monoethyl ether, from Gattefossé) and mixed in Excipial^®^ hydrocream (Galderma) (F3) [30]. Excipial^®^ hydrocream is composed of 35.5% lipid phase and contains the following excipients: water, paraffin oil, isopropyl myristate, cetearyl alcohol, glyceryl stearate, pentylene glycol, and polysorbate 20.
- Formulation 4 (F4) corresponding to the lipophilic oral solution of rapamycin (Rapamune^®^ 1 mg mL^−1^, Pfizer) directly applied to the skin. In this formulation, rapamycin was solubilized in the presence of surfactants (polysorbate 80) and Phosal 50 PG^®^ (phosphatidylcholine 50% in propylene glycol) that compose Rapamune^®^ solution [18,31,32,33,34].
- Formulation 5 (F5) corresponding to rapamycin hydro-alcoholic gel prepared by solubilizing rapamycin API in 30% (*w*/*w*) of absolute ethanol (VWR BDH Prolabo^®^) [35]. Composition of the gel is provided in Table 1.

### 2.2. Solubility of Rapamycin in Solvents

The solubility of rapamycin in liquid excipients was determined by high performance liquid chromatography (HPLC). An excess amount of rapamycin was added to 1 mL of various excipients and the mixture was incubated for 72 h at 25 °C with continuous stirring at 600 rpm. The undissolved rapamycin was removed by centrifugation at 15,000 rpm for 30 min. Thereafter, the clear supernatant was diluted and analyzed by HPLC for drug content. The solubility of rapamycin in each excipient was reported as mg mL^−1^. Experiments were performed in triplicate (*n* = 3).

### 2.3. HPLC Conditions

Assay of rapamycin was performed by UV-HPLC using a validated method [36]. Briefly, chromatographic separation was achieved by using a Dionex Ultimate 3000 coupled with a Photodiode Array Detector PDA-3000 operating between 190 to 800 nm (Thermo Fisher Scientific, Waltham, MA, USA). The chromatographic column utilized was an Interchim 250 × 4.6 mm KR-C18 (5 µm) column maintained at 40 °C. The mobile phase A consisted of mixture of water-formic acid 0.003 vol% (Merck) and mobile phase B was acetonitrile-formic acid 0.003 vol%. Acetonitrile and methanol (Chromasolv^®^) were HPLC-grade (Sigma-Aldrich, Saint-Louis, MO, USA). Elution was achieved a gradient of 50:50 to 10:90 (phase A: phase B) over 25 min. The flow rate was 1.0 mL min^−1^ and the injection volume was 50 µL. Rapamycin detection was processed at 277 nm. This method was validated according to the ICH specificities (Q2R1) and data were provided in Supplementary Materials (Section 1).

### 2.4. Measurement of Viscosity and pH

Rheological experiments were conducted to measure the viscosity of the formulations. Measurements were performed at 20 °C using rheometer RM-200 CP-4000 Plus (Lamy Rheology Instruments, Champagne-au-Mont-d’Or, France) equipped with cone-plate geometry CP-6005 (with diameter 60 mm, cone angle 0.5°, truncation 50 µm). Viscosity was measured at constant shear rate of 360 s^−1^ for 100 s. The method was validated by using 2162/9 cone-plate viscosity standard (568 mPa s) as a control (Paragon Scientific™, Birkenhead, UK). The pH determination was evaluated using a calibrated Consort P901 pH meter equipped with VWR 662-1769 pH electrode.

### 2.5. Permeation Studies

Permeation of the drug was studied through a membrane or skin, using the standardized methodology of Franz diffusion cells. These had an effective diffusion area of 1.767 cm^2^ and a receptor volume of 7.0 mL (Teledyne Hanson^®^ research, Chatsworth, CA, USA). The receptor compartment was set at 32 °C, using a circulating water bath, to mimic the skin temperature at physiological level and stirred at a speed of 400 rpm. The diffusion cells were allowed to equilibrate at 32 °C for 30 min. Then, at time zero, 0.2 g of preparation was added to the donor compartment of each Franz diffusion cell. At pre-determined time intervals, 500 µL of receptor fluid was collected and a same volume of fresh preheated medium was reintroduced into the receiver to retain the sink conditions in the system. Samples were analyzed using HPLC method to determine the amount of rapamycin diffused. All experiments were carried on 6 cells.

#### 2.5.1. In Vitro Permeation Study on Strat-M^®^

Formulations were applied to the shiny top layer of the Strat-M^®^. The receptor compartment was filled with 30:70 (*v*:*v*) filtered ethanol:phosphate buffer pH 7.4 solution (PBS, Dako^®^, Carpinteria, CA, USA).

#### 2.5.2. Ex Vivo Permeation Study on Human Skin

Dermatomed human skin (thickness 500 µm) from abdominoplasty was purchased from Proviskin (Besançon, France). Skins were obtained from human volunteers following the ethical principles and were previously approved by French Ethics Committee (N°AC 2014-2233 the 18 February 2015 and DC 2014-2227 the 13 January 2015). The receptor compartment was only filled with filtered PBS (pH 7.4). Formulations were applied to dermatomed human skin. The dermal side was in contact with PBS solution. At the end of permeation experiment, the diffusion cells were dismantled, and each sample was carefully washed in order to ensure the removal of the residual formulation from the skin surface. Skin samples were dried with a cotton swab. To verify the effectiveness of the wash procedure, the rapamycin remaining on the skin was quantified by HPLC. The epidermis was subsequently separated from the dermis by peeling with forceps without prior chemical or physical (heat) treatment. Then, each compartment was cut into small pieces and soaked in methanol for 12 h with continuous stirring at room temperature. The extraction procedure was previously validated (Supplementary Materials, Section 2). The samples extracted from skin were then centrifuged at 10,000 rpm for 15 min and analyzed by HPLC.

### 2.6. Data Analysis

Fick’s law can be considered to describe the steady-state permeation through the skin and it assumes that, under sink conditions, the drug concentration in the receiver compartment is negligible compared to that in the donor compartment [37]. The steady-state flux per unit area (J_ss_) of the drug is expressed as follow (Equation (1)):

(1) $$\mathrm{Jss}=\frac{P\times \mathrm{Cd}\times D}{e}$$

where P, C_d_ and D correspond to the partition coefficient, the donor drug concentration and the diffusion coefficient, respectively. e was the thickness controlled and given by the supplier.

Permeability coefficient (K_p_) and lag time values (T_lag_) were calculated using the pseudo steady-state from plots of cumulative penetration of rapamycin versus time. T_lag_ was calculated from linear extrapolation of the steady-state portion of the curves back to *x*-axis. The slope of the linear part of the curves (R^2^ ≥ 0.99) yielded the pseudo steady-state flux J_ss_ (µg cm^−2^ h^−1^) [37,38,39]. K_p_ was calculated according to the Equations (2)–(4):

(2) $$\mathrm{Kp}=\frac{\mathrm{Jss}}{\mathrm{Cd}}$$

The D value was calculated from the lag time (T_lag_) with the following equation:

(3) $$\mathrm{Tlag}=\frac{e²}{6\times D}$$

The P was calculated using the equation:

(4) $$\mathrm{Kp}=\frac{D\times P}{e}$$

### 2.7. Statistical Analysis

Data are presented as the mean ± standard deviation. The results were analyzed by nonparametric tests, using the Kruskal–Wallis and Mann–Whitney pairwise test for the comparison non-paired variables. A 2-tailed *p* values less than 0.05 was considered statistically significant.

## 3. Results and Discussion

### 3.1. Solubility Results

The solubility of rapamycin in the excipients/vehicles that make up the various formulations tested is given in Table 2. The results presented in Table 2 show that polar solvents such as Transcutol^®^ P and absolute ethanol provide the best solubility. Conversely, rapamycin is practically insoluble in glycerin, petroleum jelly and paraffin oils despite a lipophilic logP at 4.3. The ointment (F1) and creams (F2 or F3) composition is not conducive to solubilizing the active ingredient at the concentration studied. All else being equal, the addition of an excipient in which the active ingredient is easily soluble must have improved its solubility in the Excipial^®^, in which the Transcutol^®^ was actually added. The comparative permeation study presented below aims in part to assess the importance of this parameter.

### 3.2. Viscosity and pH

Results for the pH and viscosity of all the formulations are provided in Table 3. The range of pH of the formulations was 3.9 to 7.8, which is close to the pH of human skin. Rapamycin does not have dissociable functions within the pH range of 1–10 [40]. This parameter should therefore not affect the permeation of rapamycin. Conversely, viscosity may influence the release of drug by altering the diffusion rate from its vehicle [14]. Viscosity of the formulations ranged from 173.1 to 1299.0 mPa s and these results are discussed below.

### 3.3. In Vitro Permeation Study on Strat-M^®^

Synthetic membranes such as Stat-M^®^ represent an alternative to the use of human or animal skin because they have the advantage of providing reproducible results by limiting the lot-to-lot variability, being inexpensive and easily resourced [14,41]. Strat-M^®^ is a 300 µm thick multilayer membrane made of polyester sulfone engineered to mimic the different layers of skin. In addition, many authors have shown a strong correlation between human skin and Strat-M^®^ for both hydrophilic and lipophilic molecules [14,41,42,43,44,45]. Thus, Strat-M^®^ membranes were used in our study as a diffusion model. The process of absorption of a drug from its vehicle into the deep layers of the skin or membrane is a passive kinetic process along a concentration gradient and encompasses two main steps [46]. The first step consists in releasing the active substance from its vehicle. The permeation parameter used to evaluate this first limiting step is the partition coefficient (P), which reflects the preferential distribution of the active substance for the *stratum corneum* or the vehicle. The other limiting step corresponds to the diffusion of the active substance through the lipophilic and hydrophilic layers of the skin. This penetration step is essentially related to the physical chemical properties of the drug and factors related to the vehicle/excipients. The most relevant permeation parameters for this step are the diffusion coefficient (D) and the permeability coefficient (K_p_).

**Figure 1:**
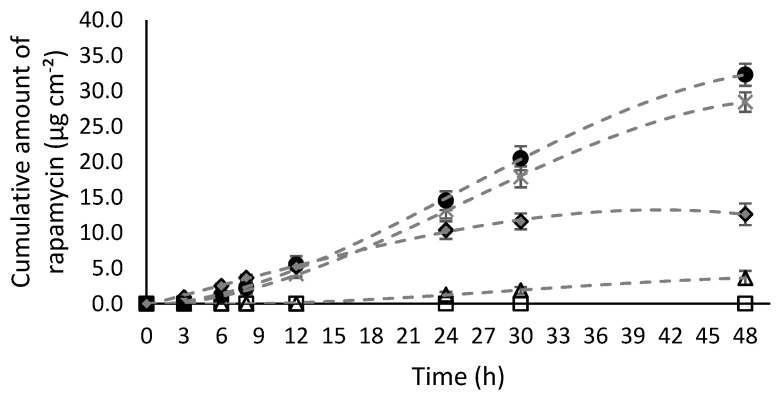
Cumulative amount of rapamycin (µg cm^−2^) diffused through Strat-M^®^ membrane during 48 h. □ corresponds to F1 (0.1% rapamycin API (active pharmaceutical ingredient) dispersed in petroleum ointment); ∆ corresponds to F2 (0.1% rapamycin API dispersed in Dexeryl^®^ cream); × corresponds to F3 (0.1% rapamycin solubilized in Transcutol^®^ P and mixed in Excipial^®^ hydrocream); ♦ corresponds to F4 (0.1% rapamycin oral solution Rapamune^®^) and ● corresponds to F5 (0.1% rapamycin hydro-alcoholic gel). Each point corresponds to the mean of 6 assays ± SD.

The permeation profiles of rapamycin through Strat-M^®^ for all formulations tested at 0.1% of rapamycin are shown in Figure 1, while their fluxes (J_ss_) and some other permeation parameters are reported in Table 4.

#### 3.3.1. Effect of Formulation Type (Hydrophilic versus Lipophilic)—Impact on Drug Release

The hydrophilic or lipophilic character of the semi-solid base influences the preferential distribution of the active ingredient for the *stratum corneum* and consequently influences the release step from formulation [47]. The formulations tested can be classified according to their physicochemical properties as bases: lipophilic monophasic (ointment F1 and oral solution F4), emulsions which are multiphase hydrophilic-lipophilic systems (creams F2 and F3) and hydrophilic monophasic (hydrogel F5). The permeation data (Table 4) show that the lipophilic character of the preparation decreases partition coefficient between the *stratum corneum* and the vehicle (P). The lowest P was obtained with the oral solution (F4) and could not be calculated for the F1 ointment. Given the very low solubility of rapamycin in water (2.6 µg mL^−1^) and its high lipophilicity (logP 4.3), the preferential distribution for *stratum corneum* is therefore lower compared to other drug release limiting formulations. Indeed, the release of lipophilic active substances from hydrophobic bases is limited, even when dissolved in the base, due to their high preferential distribution for the lipophilic excipients of the vehicle [47]. The creams (F2 and F3) are oil/water emulsions. For these predominantly hydrophilic creams, P is significantly higher than for the oral solution (F4) but remains significantly lower than a hydrophilic base such as F5. As a result, under the tested conditions, the order of P was F5 > F3 > F2 > F4 > F1. These results are in agreement with Tanaka et al. who showed through an in vitro study using a three-dimensional cultured human skin model a significantly higher percutaneous absorption with hydrogel compared to lipophilic ointment [48].

#### 3.3.2. Impact of the Physical State of the Molecule (Solubilized versus Dispersed)—Impact on Drug Permeation

As shown in Figure 1 and Table 4, the physical state of the active ingredient significantly affects the stages of absorption of the active ingredient through the Strat-M^®^. Indeed, when rapamycin is mostly dispersed rather than solubilized (F1 and F2), the concentrations found in the receiving compartment are very low for F2 and even undetectable for F1, even after 48 h of diffusion. It was therefore not possible to calculate the permeation parameters for the F1 ointment prepared from petroleum. With regard to the permeation parameters detailed in Table 4, the phase of absorption of rapamycin through the membrane represents the limiting stage of diffusion for F2. Indeed, the flux, the amount diffused, T_lag_, D and K_p_ are all significantly lower compared to other formulations using rapamycin in solubilized form. This can be explained by the poor dissolution of rapamycin, thereby increasing the lag time (T_lag_: 13.3 ± 0.8 for F2) and consequently reducing the diffusion coefficient across the membrane (D: 1.1 ± 0.07 × 10^−5^ cm^2^.h^−1^). Indeed, according to the Noyes–Whitney equation, the dissolution rate is dependent on the particle size [49]. This is why for the dispersed forms, mechanical particle-size reduction using micronization is an appropriate way to increase bioavailability by increasing the surface area of the particles [50]. Thus, as Norrenberg et al. have shown, the efficiency is higher when rapamycin is in micronized form [8].

As expected, good solubilization of rapamycin in the vehicles (F3, F4 and F5) actually improved permeation. Indeed, the cumulative quantity at 24 h for F3, F4, and F5 are between 7 to 12 times higher than that of F2. The highest flux was obtained for the hydro-alcoholic gel (F5) while the lowest was obtained with the Rapamune^®^ oral solution (F4). The presence of permeation enhancers, such as surfactants (F4) or alcohols (Transcutol^®^ P and ethanol for F2 and F5, respectively), and their concentration may increase the solubility of rapamycin in the skin and improve diffusivity across the membrane. They interact and alter the complex structure of the skin (e.g., intercellular lipid fluidization or solubilization, increasing drug solubility/partitioning in the *stratum corneum*, denaturing skin surface proteins, etc.) and thus transiently increase permeability [51,52,53]. Indeed, the high amount of surfactant in the oral solution (F4) allows rapid diffusion of the molecule across the membrane as shown by the shortest T_lag_ and the highest D. However, as previously stated, the release step represented by the P is strongly diminished or much slower with lipophilic vehicles (F2 versus the others in Figure 1).

As for the viscosity, which can act as a barrier to permeation, the values measured for each existing preparation (Table 3) do not point in this direction since, for example, the hydrogel with the highest viscosity did allow the best permeation. For this reason, although these parameters can be taken into account, in this case it seemed that their influence was minor with regard to the factors highlighted in this study.

Finally, the hydroalcoholic gel (F5) had the best permeation parameters for this concentration of active ingredient (0.1%). Indeed, as shown in Table 4, the flow, the cumulative quantity, the permeability coefficient (K_p_) as well as the partition coefficient (P) are significantly higher compared to all the formulations tested. As a result, at 0.1% rapamycin, F5 > F3 > F4 > F2 > F1.

#### 3.3.3. Effect of the Concentration of Active Ingredient in the Formulation—Impact on Thermodynamic Activity

**Figure 2:**
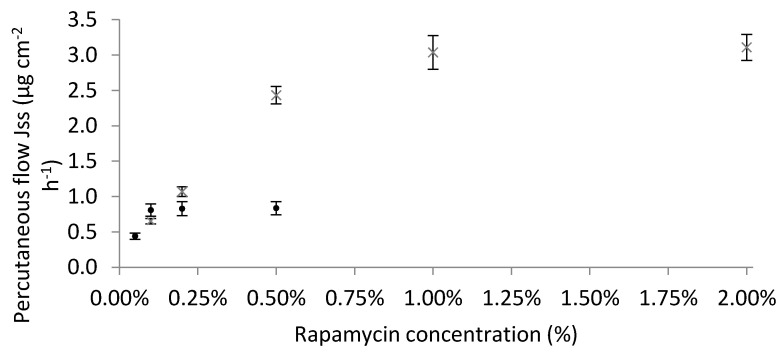
Impact of steady-state flux as a function of rapamycin concentration through Strat-M^®^ membrane. × corresponds to F3 (0.1% rapamycin solubilized in Transcutol^®^ P and mixed in Excipial^®^ hydrocream) and ● corresponds to F5 (0.1% rapamycin hydro-alcoholic gel). The percutaneous flux increases with the concentration of active ingredient until reaching a state of saturation corresponding to the maximum thermodynamic activity. Each point corresponds to the mean of 6 assays ± SD.

**Figure 3:**
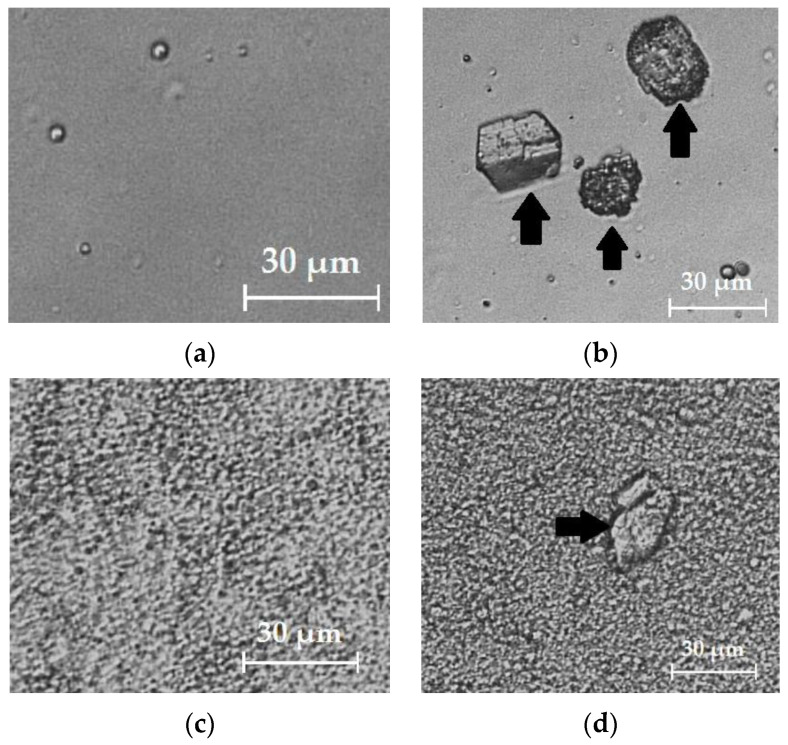
Microscopy images of formulations F3 and F5. Images were captured using an optical microscope Olympus IM coupled to a Sony camera XCD-U100CR at 924× magnification. (**a**): F5, solubilized rapamycin hydro-alcoholic gel (0.1% *w*/*w*); (**b**): F5, suspended rapamycin hydro-alcoholic gel (0.5% *w*/*w*); (**c**): F3, solubilized rapamycin Excipial^®^ cream (0.1% *w*/*w*); (**d**): F3, suspended rapamycin Excipial^®^ cream (2% *w*/*w*). Rapamycin crystals are indicated by the black arrows.

The thermodynamic activity of the drug in the vehicle depends on its concentration. Indeed, as shown in Figure 2, the results show that the percutaneous flux increases with the concentration of active ingredient until reaching a state of saturation. At saturation, the thermodynamic activity of the drug in its preparation is therefore at its maximum (α_v_ = 1) and diffusion is therefore maximal. The thermodynamic activity is based on the degree of solubility of the active principle in its vehicle. Indeed, as shown in Table 2, Transcutol^®^ P allows a higher solubility of rapamycin compared to absolute ethanol. Thus, as shown in Table 5, the maximum thermodynamic activity (α_v_ = 1) is reached for F3 at 1% rapamycin versus 0.1% for F5. The improvement in permeation for F5 compared to F3 at an active concentration of 0.1% can be also attributed to an increase in thermodynamic activity. Beyond the solubilization limit, part of the rapamycin is crystallized (Figure 3) and the diffusion is altered. At this point, the excess of active ingredient acts as a reservoir to maintain the concentration gradient over an extended period [14].

For the F1 and F2 formulations, the maximum thermodynamic activity is quickly reached considering the very low solubility of rapamycin in these vehicles (Table 2). Compared to the other formulations studied, the oral solution had the highest solubilizing power. Indeed, the solubilization limit is reached for a concentration close to 1.6% in rapamycin, i.e., about 2 and 16 times greater than F3 and F5, respectively (Table 2). Thus, the 0.1% oral solution has a low thermodynamic activity because the dissolved quantity is not close to the saturating concentration, which makes the skin permeability less favorable compared to F3 or F5. Thus, drug partition decreases with increasing vehicle solubilizing power [54].

For the same formulation, the increase in concentration does not influence the T_lag_ and therefore the diffusion coefficient (D) because these parameters depend on the vehicle used. On the other hand, as the concentration increases, the drug partition (P) decreases, making the drug release more and more limiting and consequently saturating the skin flow.

### 3.4. Ex Vivo Permeation Study on Human Skin

The effectiveness of the wash procedure was confirmed by HPLC. Indeed, the rapamycin remaining from the skin surface was found to be below the limit of quantification of the analytical method.

It should be noted that the selected PBS-containing recipient compartment does not correspond to the sink conditions because the active substance is poorly soluble in it (i.e., 1.6 ± 0.9 µg mL^−1^). However, as we carried out a comparative study by setting identical experimental conditions for all, the relative differences found between the tests should be effective.

**Figure 4:**
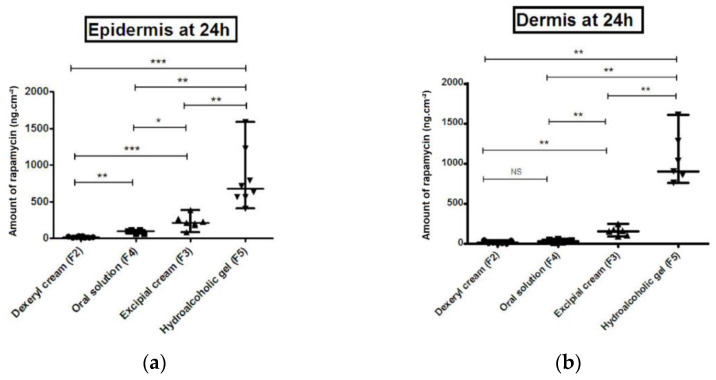
Cumulative amount of rapamycin per unit area (ng cm^−2^) over 24 h from different formulation through epidermis (**a**) and dermis (**b**) of human skin. Data are presented as the median and range. NS: non-significant, * *p* < 0.05, ** *p* < 0.01, *** *p* < 0.001.

For a same concentration of active ingredient (0.1%), the results of ex vivo studies on human skin are in agreement with those of previous in vitro studies on Strat-M^®^ synthetic membrane. Indeed, as shown in Figure 4, the quantity diffused into the skin is systematically significantly higher when rapamycin is in the soluble state. Indeed, the crystalline state of rapamycin (F2 formulation) limits the diffusion of the active ingredient through the superficial layers of the skin. Conversely, the soluble state of rapamycin allows a significantly higher diffusion through the skin. The lipophilic character of the oral solution as shown above limits the diffusion of rapamycin. Indeed, the amount diffused into the dermis for the F2 and F4 formulations does not differ significantly (Figure 4b). On the other hand, the amount diffused into each compartment differs significantly between F3 and F5 formulations. Indeed, the quantity diffused into the epidermis and dermis remains significantly higher with the F5 hydrophilic gel than with the F3 cream because at this concentration the thermodynamic activity is maximal for F5 (α_v_ = 1). As result, at 0.1% rapamycin, F5 > F3 > F4 > F2 > F1 as before.

## 4. Conclusions

In conclusion, this work reports, for the first time, a comparative study of the existing formulations used for the management of facial angiofibromas based on in vitro/ex vivo permeability with respect to the composition of the vehicles. This study highlights the importance of the nature of the formulation used (hydrophilic versus lipophilic), the importance of having rapamycin solubilized and at a concentration close to the solubility limit in order to ensure maximum thermodynamic activity and to promote optimal cutaneous bioavailability. Thus, the present study provides essential tools for the development and optimization of topical rapamycin formulations using a pharmaceutical and biopharmaceutical approach. It could serve as a reference for prediction of in vivo cutaneous absorption and thus for clinical efficacy.

In the light of the relative behavior of the existing formulations described in the literature, it appeared that at the same usual concentration (i.e., 0.1%), hydrogel-type vehicles seem to have produced better release and permeation than the others. Therefore, in the present situation, gel-based formulations seem to be recommended. On the other hand, the use of solubilizing agents like Transcutol^®^ P in vehicle-based emulsions also improved permeation. That is why combining hydrogel and Transcutol^®^ is worth to be tested. Thus, we are going to investigate the role of Transcutol^®^ in hydrogels and the approach will be extended to other potential drug candidates that could be used in topical formulations, i.e., for instance, everolimus or tacrolimus.

## Figures and Tables

**Table 1:** Composition of rapamycin hydro-alcoholic gel formulation (F5).

Components	F5 (% *w*/*w*)
Carbopol-974P	0.75
Glycerin	15
Absolute ethanol	30
α-tocophérol	0.2
Urea	1
Water	Quantity sufficient for 100

**Table 2:** Rapamycin solubility in the solvents used and formulations at 25 °C.

Excipients	Rapamycin Solubility (mg mL^−1^) ^1^
Transcutol^®^ P	144.8 ± 4.5
Ethanol absolute	86.1 ± 4.0
Oral solution Rapamune^®^	15.7 ± 0.2
Glycerin	<0.01
Petroleum oil	<0.01
Paraffin oil	<0.01

^1^ Data are represented as mean ± standard deviation. All measurements are done in triplicates (*n* = 3).

**Table 3:** pH and viscosity of rapamycin formulations.

Formulation	pH	Viscosity (mPa s) at 20 °C
F1	3.9	960.2
F2	7.8	173.1
F3	5.2	283.1
F4	5.5	301.0
F5	6.7	1299.0

**Table 4:** Permeation parameters for the 0.1% rapamycin formulations.

Formulation	J_ss_ (µg cm^−2^ h^−1^) ^1^	K_p_ × 10^−4^ (cm h^−1^) ^1^	T_lag_ (h) ^1^	D × 10^−5^ (cm^2^ h^−1^) ^1^	P _skin/vehicle_^1^	Cumulative Amount Diffused at 24 h (µg cm^−2^) ^1^
F1	ND	ND	ND	ND	ND	<LOD
F2	0.1 ± 0.03 °*^§^	1.2 ± 0.3 °*^§^	13.3 ± 0.8 °*^§^	1.1 ± 0.07 °*^§^	0.3 ± 0.04 °*^§^	1.3 ± 0.4 °*^§^
F3	0.7 ± 0.05 ^φ†^	6.8 ± 0.5 ^φ†^	5.1 ± 0.5 ^φ^	3.0 ± 0.3 ^φ^	0.7 ± 0.05 ^φ†^	12.8 ± 1.2 ^φ†^
F4	0.5 ± 0.06 ^◊^	5.1 ± 0.6 ^◊^	1.1 ± 0.3 ^◊^	12.0 ± 1.2 ^◊^	0.1 ± 0.01 ^◊^	10.0 ± 1.2 ^◊^
F5	0.8 ± 0.08	7.9 ± 0.8	4.8 ± 0.6	3.2 ± 0.4	0.8 ± 0.06	15.3 ± 1.5

^1^ Data are represented as mean ± standard deviation of 6 assays (*n* = 6). ° F2 versus F3, * F2 versus F4, ^§^ F2 versus F5: *p* < 0.05; ^φ^ F3 versus F4, ^†^ F3 versus F5: *p* < 0.05; ^◊^ F4 versus F5: *p* < 0.05. ND: not determined; LOD: limit of detection; J_ss_: steady-state flux; K_p_: permeability coefficient; T_lag_: lag time; D: diffusion coefficient (thickness set at 300 µm); P_skin/vehicle_: partition coefficient between skin and vehicle.

**Table 5:** Effect of rapamycin concentration on permeation parameters from formulations F3 and F5.

Rapamycin Concentration	Formulation	0.05%	0.1%	0.2%	0.5%	1%	2%
Thermodynamic activity (α_v_)	F3	0.05	0.1	0.2	0.5	1	1
	F5	0.5	1	1	1	NT	NT
J_ss_ (µg cm^−2^ h^−1^) ^1^	F3	NT	0.7 ± 0.05 ^†^	1.1 ± 0.07 ^†^	2.4 ± 0.1 ^†^	NS	
						3.0 ± 0.2	3.1 ± 0.2
	F5	0.4 ± 0.05	^NS^			NT	NT
			0.8 ± 0.08	0.8 ± 0.1	0.8 ± 0.09		
K_p_ × 10^−4^ (cm h^−1^) ^1^	F3	NT	6.8 ± 0.5 ^†^	5.4 ± 0.3 ^†^	3.9 ± 0.1 ^†^	2.9 ± 0.4	1.6 ± 0.07
	F5	8.7 ± 0.9	7.9 ± 0.8	4.4 ± 0.7	1.7 ± 0.2	NT	NT
T_lag_ (h) ^1^	F3	NT	^NS^				
			5.1 ± 0.5	4.8 ± 0.4 ^†^	4.7 ± 0.3 ^†^	4.7 ± 0.4	4.7 ± 0.3
	F5	^NS^				NT	NT
		5.3 ± 0.5	4.8 ± 0.6	5.4 ± 0.5	5.4 ± 0.5		
D × 10^−5^ (cm^2^ h^−1^) ^1^	F3	NT	^NS^				
			3.0 ± 0.3	3.2 ± 0.2 ^†^	3.2 ± 0.2 ^†^	3.2 ± 0.3	3.2 ± 0.2
	F5	^NS^				NT	NT
		2.9 ± 0.3	3.2 ± 0.4	2.8 ± 0.3	2.8 ± 0.3		
P _skin/vehicle_^1^	F3	NT	0.7 ± 0.05 ^†^	0.5 ± 0.05	0.4 ± 0.02 ^†^	0.3 ± 0.04	0.1 ± 0.01
	F5	1.0 ± 0.09	0.8 ± 0.06	0.4 ± 0.02	0.2 ± 0.02	NT	NT
Cumulative amount diffused (µg cm^−2^) at 24 h ^1^	F3	NT	12.8 ± 1.2 ^†^	20.6 ± 1.1 ^†^	48.0 ± 2.9 ^†^	^NS^	
						59.4 ± 4.9	61.2 ± 4.5
	F5	8.1 ± 1.0	^NS^			NT	NT
			15.3 ± 1.5	16.8 ± 1.8	16.8 ± 1.5		

^1^ Data are represented as mean ± standard deviation of 6 assays (*n* = 6). ^†^ F3 versus F5 (for the same concentration): *p* < 0.05; NS: non-significant (for the same formulation); NT: not tested. J_ss_: steady-state flux; K_p_: permeability coefficient; T_lag_: lag time; D: diffusion coefficient (thickness set at 300 µm); P_skin/vehicle_: partition coefficient between skin and vehicle.

## Supplementary Materials

The following are available online at https://www.mdpi.com/1999-4923/12/11/1060/s1. Table S1: Linearity, LOD and LOQ of the HPLC method, Table S2: Accuracy and Precision of the HPLC method.
