# Supplementary material for: Comparison of the In Vitro and Ex Vivo Permeation of Existing Topical Formulations Used in the Treatment of Facial Angiofibroma and Characterization of the Variations Observed
Source: Pharmaceutics. 2020 Nov 7;12(11):1060. doi: 10.3390/pharmaceutics12111060 (PMC7694993; doi:10.3390/pharmaceutics12111060)
Supplement: Supplementary file 1 [file pharmaceutics-12-01060-s001.pdf]

pharmaceutics

Supplementary Materials: Comparison of the In Vitro and Ex Vivo Permeation of Existing Topical Formulations Used in the Treatment of Facial Angiofibroma and Characterization of the Variations Observed

Guillaume Le Guyader, Bernard Do, Victoire Vieillard, Karine Andrieux and Muriel Paul

1. High-Performance Liquid Chromatography: Analytical Method and Validation

1.1. Method

HPLC method was validated for linearity, accuracy, precision, limit of detection and limit of quantification, in accordance with the International Consensus on Harmonization guidelines (ICH Q2R1).

Linearity, Limit of Detection and Limit of Quantification:

Linearity of this method was evaluated by spiking eight concentrations of rapamycin (0.5, 1, 2, 3, 4, 6, 8 and 10 µg mL^{-1}) in methanol and injecting into the chromatographic instrument. A calibration curve was plotted for peak area versus drug concentration and a coefficient of determination (R^2) was determined by linear least-square regression analysis.

The limit of detection (LOD) was calculated as 3.3 (σ/S), where σ is the standard deviation of the y-intercept and S is the slope of the calibration curve. Similarly, the limit of quantification (LOQ) was calculated as 10 (σ/S).

Precision and Accuracy:

The precision of the method was assessed as the intra-day precision (repeatability) and the inter-day precision (intermediate precision) and given as relative standard deviation (RSD). To this end, intra-day precision was determined using 18 samples with high concentration levels (6 samples of 10 µg mL^{-1}), intermediate concentration levels (6 samples of 6 µg mL^{-1}), and low concentration levels (6 samples of 1 µg mL^{-1}). These samples were prepared and injected into the chromatography system on the same day. The inter-day precision was estimated from the analysis of standard solutions at three concentration levels (1, 6, and 10 µg mL^{-1}) over 3 successive days. Six samples of each concentration were prepared and analyzed daily.

Accuracy was carried out with calculation of the average recovery value (%) by analyzing six standard solutions at three concentration levels representing the high (10 µg mL^{-1}), the intermediate (6 µg mL^{-1}), and the low (1 µg mL^{-1}) level of the linear range of calibration curve.

1.2. Results

As shown in Tables S1 and S2, the method can provide quantification of rapamycin in the linearity range with good precision and accuracy. The obtained results are in accordance with ICH guideline.

Table S1. Linearity, LOD and LOQ of the HPLC method.

Figure of merit	Value
Linear range (μg mL^{-1})	0.5–10
Slope (a)	2.510
Intercept (b)	0.067
Determination coefficient (R^2)	0.9963
Limit of Detection (LOD)	0.023 μg mL^{-1}
Limit of Quantification (LOQ)	0.070 μg mL^{-1}

Table S2. Accuracy and Precision of the HPLC method.

Concentration tested (μg mL^{-1})	Accuracy-Recovery %	RSD Precision (%)	
		Intra-day	Inter-day
1	100.1 ± 1.2	0.8	1.2
6	99.1 ± 0.3	0.1	0.3
10	99.6 ± 0.6	0.2	0.7

2. Validation of Human Skin Extraction Procedure

2.1. Method

A validated extraction procedure was tested to evaluate the ability of this method to recover the rapamycin deposited during ex vivo permeation experiments. Thereby, dermatomed human skin samples (n = 3) were spiked with a known amount of rapamycin in methanol (10 mg mL^{-1}). Methanol facilitated rapamycin skin deposition and was subsequently evaporated. Then, skin samples were cut into small pieces and soaked in methanol for 12 h with continuous stirring at room temperature. At last, skin samples were centrifuged at 10,000 rpm for 15 min and analyzed by HPLC. The amounts of rapamycin recovered were compared to the amounts initially applied.

2.2. Results

Regarding to the validated procedure, 80.2 ± 4.3% ($n = 3$) of rapamycin was recovered during the extraction method from total human skin. The extraction method was therefore considered as suitable for rapamycin extraction in the ex vivo permeation experiments.
